# Commentary: Family life in lockdown

**DOI:** 10.3389/fpsyg.2025.1454643

**Published:** 2025-06-19

**Authors:** Leo Van Hove

**Affiliations:** FIRE Research Group and Department of Applied Economics (APEC), Vrije Universiteit Brussel, Brussels, Belgium

**Keywords:** household division of labor, gender, gender identity, LGBT, same-sex couples, lockdown, COVID-19

## 1 Introduction

Biroli et al. (2021) analyze how COVID-19 lockdowns affected patterns of activity within households in Italy, the UK, and the US. They do so based on data from self-administered surveys among adults who indicated that they were cohabiting with their partner. Biroli et al. find that sharing of most duties increased, in particular for childcare.

As the authors report on their results in terms of which gender does more, or less, of a certain task, the reader gets the impression that their samples consist solely of different-sex couples. However, upon analyzing the questionnaire and inquiring with the authors, it became clear that they only asked respondents about their own gender. In the analysis, the authors simply attributed the opposite gender to the partner.

Biroli et al. thus risk having the gender of partners wrong, as their samples may contain same-sex couples. Given that 5.6% of US adults identified as lesbian, gay, bisexual or transgender (LGBT) in 2020 (Gallup, 2021a), at first sight the potential bias resulting from not filtering out same-sex couples would seem substantial. Below I try to come up with more precise estimates. But first I briefly discuss the set-up of Biroli et al.'s research.

## 2 Biroli et al.'s data collection

Biroli et al. ran their surveys in April 2020, during the first COVID-19 wave. For the surveys in the UK and the US, they used an online tool that stratifies samples across age, sex, and ethnicity. The Italian sample is less representative, as participants were recruited mainly through social media.

Crucially, only one member of each couple filled out the questionnaire (Biroli et al., 2021, p. 4, footnote 10), and reported both about their own situation as well as that of their partner. The problem is that, as indicated in the Introduction, respondents were not asked about their partner's gender. This was confirmed to me by the corresponding author, who explained that when they did the analysis, they attributed the opposite gender to the respondent's partner.

## 3 The resulting bias

This said, on closer scrutiny, it would appear that the bias resulting from the inclusion of same-sex couples is, all in all, limited. To start with the US, as mentioned, according to Gallup data based on more than 15,000 interviews, 5.6% of US adults (aged 18 and older) identified as LGBT in 2020 (Gallup, 2021a). This tallies almost perfectly with the 5.5% estimate proffered by the Williams Institute for 2020–2021 (Flores and Conron, 2023, p. 1).

As can be seen in Table 1, of the LGBT individuals in the Gallup data 16.7% were married to a same-sex spouse or were living with a same-sex partner. Hence, Biroli et al.'s approach risks getting the gender of the respondent's partner wrong in (only) 0.94% of their US households.

**Table 1:** Marital status of US LGBT adults.

**Marital status**	**%**
Married to opposite-sex spouse	11.4
Married to same-sex spouse	9.6
Living with opposite-sex domestic partner	9.2
Living with same-sex domestic partner	7.1
Single/never married	50.5
Separated	2
Divorced	7.5
Widowed	2.5
No opinion	0.4

Source: Gallup (2021b).

However, one could argue that since Biroli et al. (2021, p. 1) refer to “gendered patterns of intra-household cooperation norms,” they should have concentrated fully on heterosexual couples. In other words, it could be argued that they should also have left out of their analysis LGBT individuals who are living with an opposite-sex partner (as is the case for a fair share of bisexuals). Indeed, gender norms might be more flexible in such couples. From this perspective, the share of US couples that are potentially problematic amounts to 2.09%—if Biroli et al.'s sample is representative of the general population (the sum of the first four categories in Table 1 is 37.3%; to be multiplied by 5.6%.).

For the UK, the available statistics are less detailed. According to the Annual Population Survey 2020, overall 3.1% of the population aged 16 years and over identified as lesbian, gay or bisexual in 2020 (Office of National Statistics, 2022). An additional 0.7% indicated “other” (Office of National Statistics, 2023a).

The data also show that 23.7% of LGB individuals were either married or in a civil partnership. Adding the “other” category increases the share to 24.6%. Unfortunately, the survey does not contain data on LGB(T) individuals who are cohabiting with a same-sex partner without being married or being in a civil partnership. Hence, when it comes to the measurement error in Biroli et al.'s assessment of the gender of the partners of their UK respondents, one can calculate only a lower limit; namely 0.73%−0.92% (23.7% *times* 3.1%−24.6% *times* 2.7%).

The Census 2021 for England and Wales (Office of National Statistics, 2023b) does specify how many individuals are cohabiting with a same-sex partner without being married or being in a civil partnership. This source (thus) yields a slightly higher estimate than the 0.73% of the Annual Population Survey 2020, namely 1.00% (0.33% married or in a civil partnership + 0.67% cohabiting) [the censuses for Scotland and Northern Ireland do not distinguish between same- and opposite-sex marriages; Scotland's Census, 2023; Northern Ireland Statistics and Research Agency (NISRA), 2023].

Finally, for Italy there would appear to be even fewer statistics (ISTAT, 2022, 2023). But an international survey by IPSOS (2021, p. 9) suggests lower measurement errors: in Italy 6% of adults identified as non-heterosexual in 2021, compared to 10% in the UK and 11% in the US.

## 4 Conclusion

Upon scrutiny, it would appear that the bias resulting from the non-exclusion of same-sex couples is, all in all, contained. I show that Biroli et al.'s heteronormative approach risks getting the gender of the respondent's partner wrong in 0.94% of their US households, in upwards of 0.73%−1.00% of their UK couples, and in probably a lower proportion of their Italian sample. If one argues that Biroli et al. should have left out of their analysis not only LGBT individuals who are living with a same-sex partner but also those who are cohabiting with a partner of the opposite sex, then the share of US couples that are potentially problematic amounts to 2.09%.

Biases of this magnitude are unlikely to substantially alter Biroli et al.'s conclusions. Still, it is a limitation of the data that readers should be (made) aware of.
